# Supplementary material for: ProteinShader: illustrative rendering of macromolecules
Source: BMC Struct Biol. 2009 Mar 30;9:19. doi: 10.1186/1472-6807-9-19 (PMC2672931; doi:10.1186/1472-6807-9-19)
Supplement: Additional file 1 — ProteinShader program without source code. This compressed file contains the complete ProteinShader program including associated libraries, but no source code. A README.txt file gives an overview of the ProteinShader distribution, and the index.html file in the help subdirectory has directions on getting started with the program as well as a set of tutorials. [file 1472-6807-9-19-S1.zip › ProteinShader-beta-0_9_4-binary/help/development-notes.html]

ProteinShader: Development Notes


# Development Notes for the ProteinShader Program


♦ Home


♦ Download


♦ Getting Started

Choose a Link:

Installation

Running

Troubleshooting

PDB Files


♦ Tutorials

Choose a Link:

List of Proteins

Style Options

Saving Images


♦ General Controls

Choose a Link:

Menu Bar

Keyboard Shortcuts

Mouse Motion

Motion Panel

Antialiasing


♦ Cartoon Controls

Choose a Link:

Control Panels

Selection Panel

Radio Buttons

Decorations Panel

Adding Textures

Color Panel

Visibility Panel

Side Chains Panel


♦ Atom Controls

Choose a Link:

Control Panels

Selection Panel

Radio Buttons

Color Panel

Visibility Panel

Scale Panel

Tiling Panel


♦ Developers

Choose a Link:

Compiling

Running

Code Docs

Developer Links


## Compiling

- ### Compiling Java with Ant

  The ProteinShader program is compiled with Ant (Another Neat Tool).
  Ant uses the build.xml and build.properties files that are included with
  the source code distribution of the ProteinShader program (the source
  code is in the src subdirectory). Ant was originally developed by
  James Duncan Davidson, and is now an Apache open source community
  project. Most Linux, Macintosh OS X, and Windows XP machines with the
  Java SDK installed probably already have Ant, but if not, Ant can be
  downloaded for free from the Apache Ant Project web page at
  http://ant.apache.org. If you
  are not sure if Ant is already installed, type "ant -version"
  on the command line in a Terminal window (Linux or Macintosh OS X) or
  a Command Prompt window (Windows XP). If a message including something
  like "command not found" is printed, you probably need to
  download and install Ant.

  To compile the Java source code in the src directory, change into
  the ProteinShader directory (where build.xml is found), and type
  "ant" on the command line. After placing the compiled code
  into the bin directory, Ant will use the compiled code to create a
  runnable JAR file, ProteinShader.jar, which will also be placed in
  the bin directory. By giving target arguments to Ant, Ant can be used
  to create new Javadoc html for the ProteinShader API or to clean up
  unwanted files, as shown in the following list of commands, where the
  "%" indicates the command line prompt:

  - #### % ant

    The main target will compile the ProteinShader program and then
    package it into a runnable JAR file (may take a few to several
    seconds).

  - #### % ant compile

    The compile target compiles the source code in the src directory
    and places it in the bin directory (may take a few to several
    seconds).

  - #### % ant compress

    Uses the compiled code to create a runnable JAR file (so compile
    is a dependency that would get called automatically if compress
    is used). The manifest file will include Class-Main and
    Class-Path so that the JAR file is runnable.

  - #### % ant javadoc

    Extracts all public comments in the Java source code and creates
    the Javadoc html for the ProteinShader API , which is placed in
    the docs directory.

  - #### % ant clean

    Calls on the clean.classes and clean.jar targets. The entire
    bin directory is not deleted because there are dynamic libraries
    that need to remain there (".dll", ".jnilib",
    and ".so" files needed by jogl.jar).

  - #### % ant clean.classes

    Removes org, the subdirectory of bin that holds the
    ".class" files.

  - #### % ant clean.jar

    Removes the runnable JAR file in the bin directory.

  The name=value pairs for properties used in the build.xml file
  are specified in the build.properties file to make them easy to
  change. For example, to use a different source code directory,
  "src=src" in the build.properties file could be changed
  to something like "src=src2". Alternatively, the property
  could be specified by using a command line argument of the form
  'ant -Dsrc="src2"'.

  Ant is much more powerful and flexible than the shell scripts I
  used to use. If you are not familiar with Ant and wish to learn more
  about it, I recommend "Ant: The Definitive Guide" by Steve
  Holzner, Second Editon, Oreilly
  (
  www.oreilly.com/catalog/anttdg2).

- ### Vertex and Fragment Shaders

  To modify the graphics pipeline for custom lighting and texture mapping
  effects, the ProteinShader program uses small programs, known as vertex
  and fragment shaders, that are written using the OpenGL Shading
  Language. The vertex and fragment shaders (which always come in
  pairs) can be found in the shaders directory.
  The vertex and fragment shader pairs are compiled and linked by
  making calls to OpenGL, which runs over on the graphics card
  (*i.e.*, shader binaries are not created and stored in advance,
  they are generated on demand for use with the particular graphics card
  that is present on the computer).

  When the ProteinShader program starts up and first initializes the
  OpenGL state, a Java class called ShaderManager will ask an instance
  of the ShaderFactory class to read in the vertex and fragment shader
  files as long strings, and then pass those strings to the appropriate
  OpenGL command for compiling and linking. If a problem occurs, the
  information on the OpenGL error will be placed into a Java
  ShaderException. The ShaderFactory will compile and link as many
  shaders as it can, and afterwards any ShaderExceptions that occurred
  will be thrown upwards to the GUI so that the informtion can be
  displayed in a dialog box. The ProteinShader program will not exit.
  Instead it will use OpenGL's built-in lighting as a fall back
  position.


## Running

- ### Starting ProteinShader with Ant

  The Ant build tool used for compiling can also be used to start the
  program in one of two possible modes as shown in the following
  commands, where "%" indicates the command line prompt:

  - #### % ant run

    Starts the ProteinShader program on another fork. The location
    of the dynamic libraries needed for JOGL (Java Bindings for
    OpenGL) is explicitly given to the java command. The program
    operates in its normal mode, where OpenGL display lists are
    used to cache reusable geometry over on the graphics card,
    which is much faster than calling on Java drawing routines
    every frame ("reusable geometry" here means the collection of
    vertices that define spheres, cylinders, ribbons segments, and
    tube segments).

  - #### % ant run-slow

    Same as run, except that the command line argument
    "-DrenderOnTheFly=true" is included. To speed up
    rendering, the ProteinShader program normally caches geometry
    in the form of OpenGL display lists, and then executes the
    display lists when rendering. However, if renderOnTheFly is
    set to true, the program will instead call on Java drawing
    classes when rendering spheres, ribbon segments, and tube
    segments.

  The only reason for using the alternate run-slow mode is
  to determine how much of a performance increase is obtained
  by using OpenGL display lists (by comparing the normal and alternate
  mode for frames per second rendered during a constant rotation). If
  Ant is not installed, this alternate mode could be activated by adding
  "-DrenderOnTheFly=true" to the run.bat or run.sh script
  discussed in the 
  starting the program section.

  On a 2.6 GHz pentium 4 machine with a three year old NVIDIA
  FX5500 graphics card (an entry-level card) with 256 MB VRAM, caching
  geometry in advance resulted in about a 4-fold increase in frames
  per second during a constant rotation. On an iMac 2.16 GHz Intel
  Core 2 Duo machine with a one year old ATI Radeon X1600 graphics
  card (a mid-level card) with 256 MB VRAM, caching geometry resulted
  in even greater performance differences (13-fold for tubes, 9-fold
  for ribbons, and 8-fold for spheres).

  The difference in rendering speed for caching geometry rather than
  rendering on-the-fly will likely vary quite a bit depending on the
  graphics card that is in use. If you are interested in this type of
  comparison, a technical issue that you should be aware of is that on
  most machines the maximum rendering speed will only be obtained if the
  window with the image has the focus (clicking on the canvas will make
  sure that its window has the focus). Also, any other programs should
  be closed when doing performance testing.


## Code Documentation

- ProteinShader API

  The ProteinShader API (Application Programming Interface) is
  generated by the Javadoc tool, which extracts comments from the
  source code and generates the documentation in an HTML format.
  If the source code is modified and recompiled, the Javadoc can be
  updated by typing "ant javadoc" on the command line, as
  discussed further above in the section on
  Compiling Java with Ant.

- ### Overview of Java Packages

  The ProteinShader program is organized into four major packages:
  structure, gui, graphics, and math. The purpose and most important
  classes for each package are summarized in the list right below.

  - #### structure package

    The structure package holds the classes that store information
    from a Protein Data Bank file. Class Structure serves as the
    top-level container for objects that hold numeric and
    descriptive information on the three-dimensional structure of
    a protein: Model, Chain, Region (Loop, Helix, and BetaStrand),
    Residue (AminoAcid, Heterogen, and Water), and Drawable (Atom,
    Bond, and Segment).

    The most important subpackages are structure.io (includes
    StructureReader, PDBStructureReader, and PDBLineParser),
    structure.visitor (includes Visitor, Visitable, ModifierVisitor,
    and VisbilityVisitor), and structure.factory (includes
    SegmentFactory and BetaStrandFactory).

  - #### gui package

    The gui package holds all of the Java AWT and Swing components
    and their associated listener classes, including class Renderer,
    which is registered as a listener for the GLCanvas object that
    serves as a drawing surface. The ProteinShader class has the
    method main(), which creates an instance of the ProteinShaderGUI
    class and sets it visible.

    The most important subpackages are gui.components and
    gui.listeners. The gui.components package contains the menu
    bar, menu items, and control panels associated with the main
    GUI window. The gui.listeners package contains listener
    factories with methods to create listeners for the gui
    components. Typically, each component class has a
    corresponding listener factory. For example, the class
    gui.components.DecorationsPanel has a corresponding listener
    factory, gui.listeners.DecorationsPanelListenerFactory, which
    makes all of the listeners needed by the DecorationsPanel.

  - #### graphics package

    The graphics package holds the actual drawing classes that
    render tubes, ribbons, spheres, and cylinders. The
    StructureToGraphics class, which is located in the
    graphics.adapter subpackage, plays a key role in managing the
    use of the drawing classes (Tube, Ribbon, FrenetFrames,
    Cylinder, and Sphere) and has classes for managing OpenGL
    display lists, which are used for caching geometry over on the
    graphics card. The graphics package also has classes for
    managing textures (Texture, TextureManager, and
    TextureFactory) and shaders (ShaderProgram, ShaderManager, and
    ShaderProgramFactory).

  - #### math package

    The key classes in the math package are Hermite, Quaternion,
    and LocalFrame. These classes are needed for creating
    three-dimensional tubes and ribbons.


## Developer Links

- Java 1.5 API
- OpenGL
- OpenGL Shading Language Tutorial
- JOGL (Java Bindings for OpenGL)
- JOGL JSR-231 API (nightly build)


---

|  |  |  |  |
| --- | --- | --- | --- |
| Hosted by |  |  |  |

Webmaster: Joe Weber,
joe.weber AT alumni.duke.edu
  
Web site last updated: December 30th, 2008
  
© 2007–2008 Joseph R. Weber
